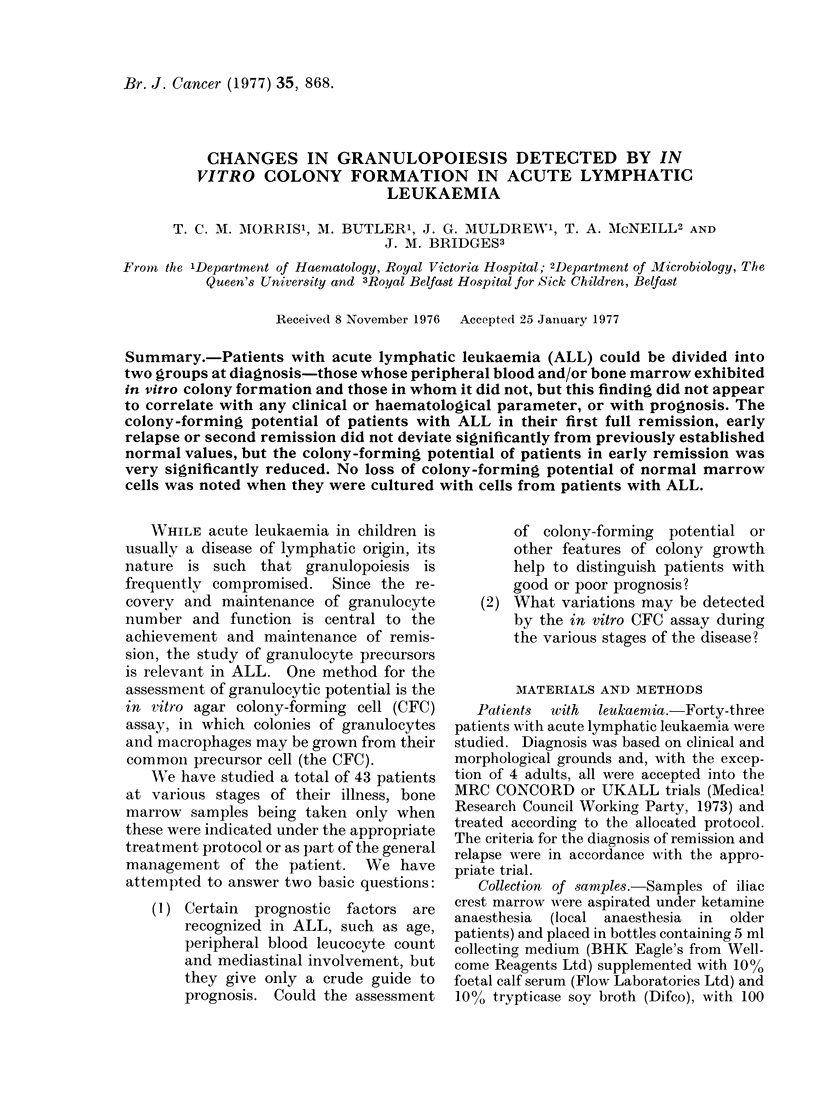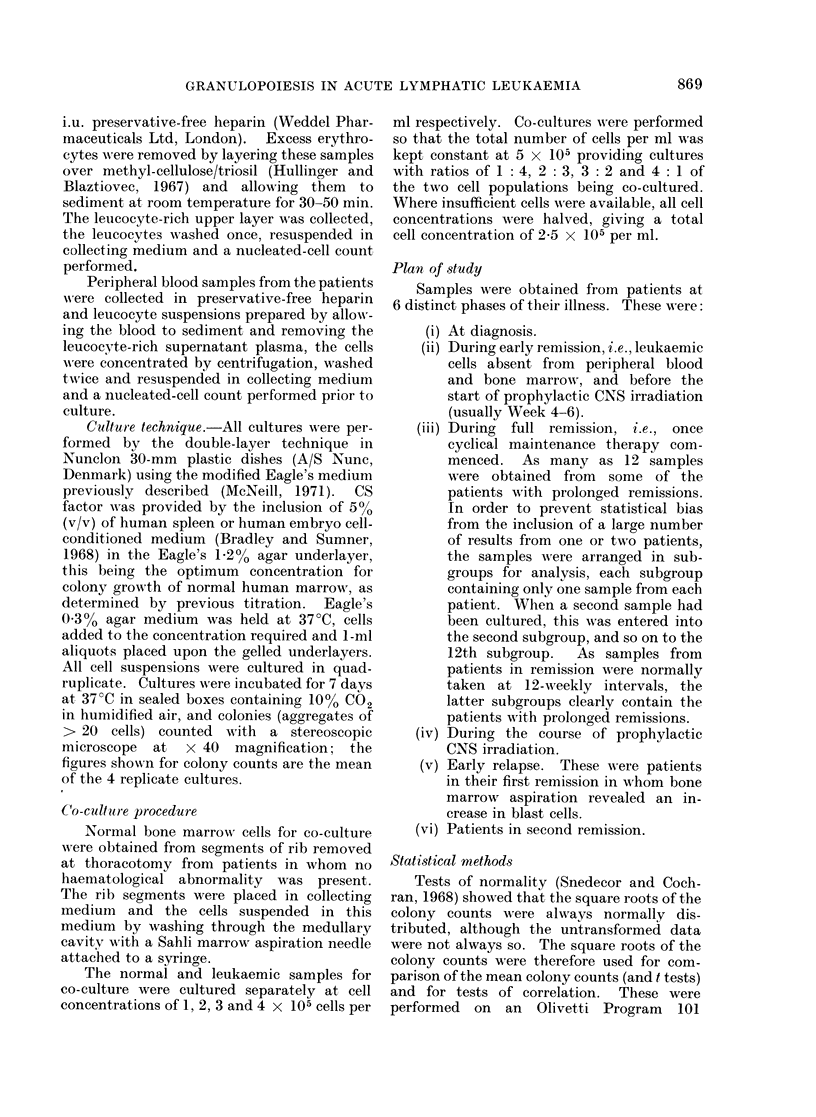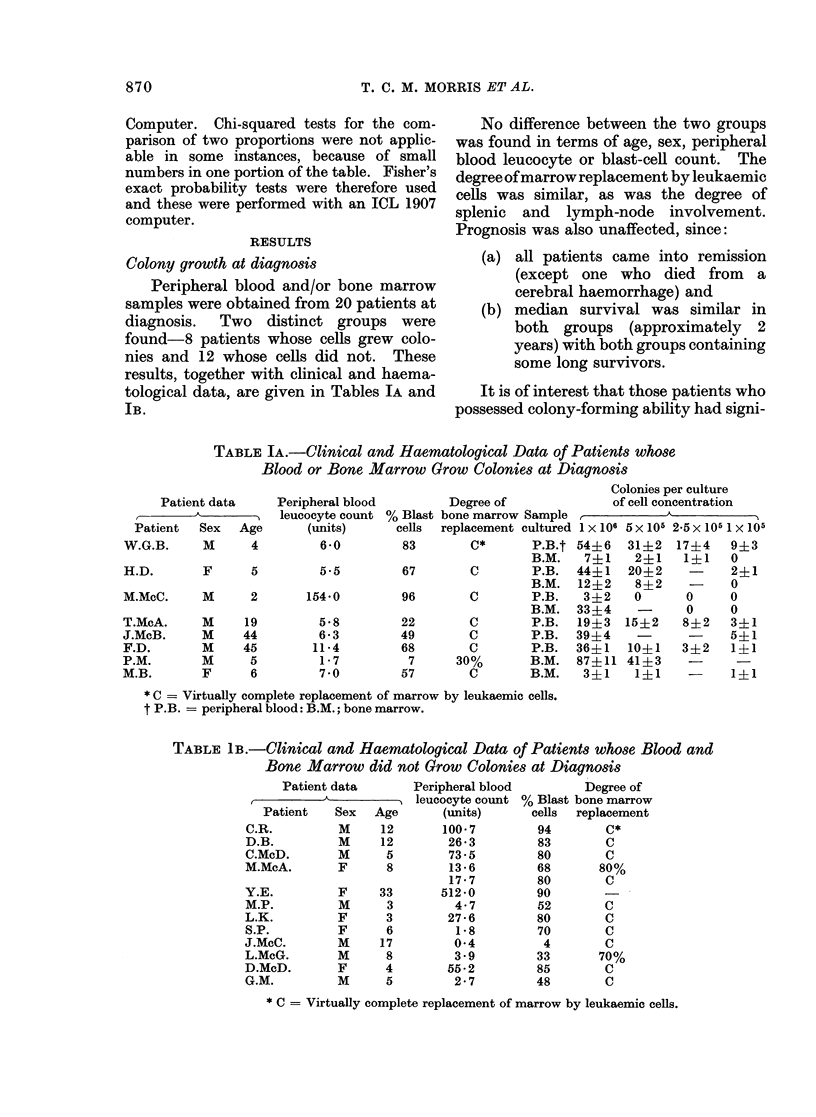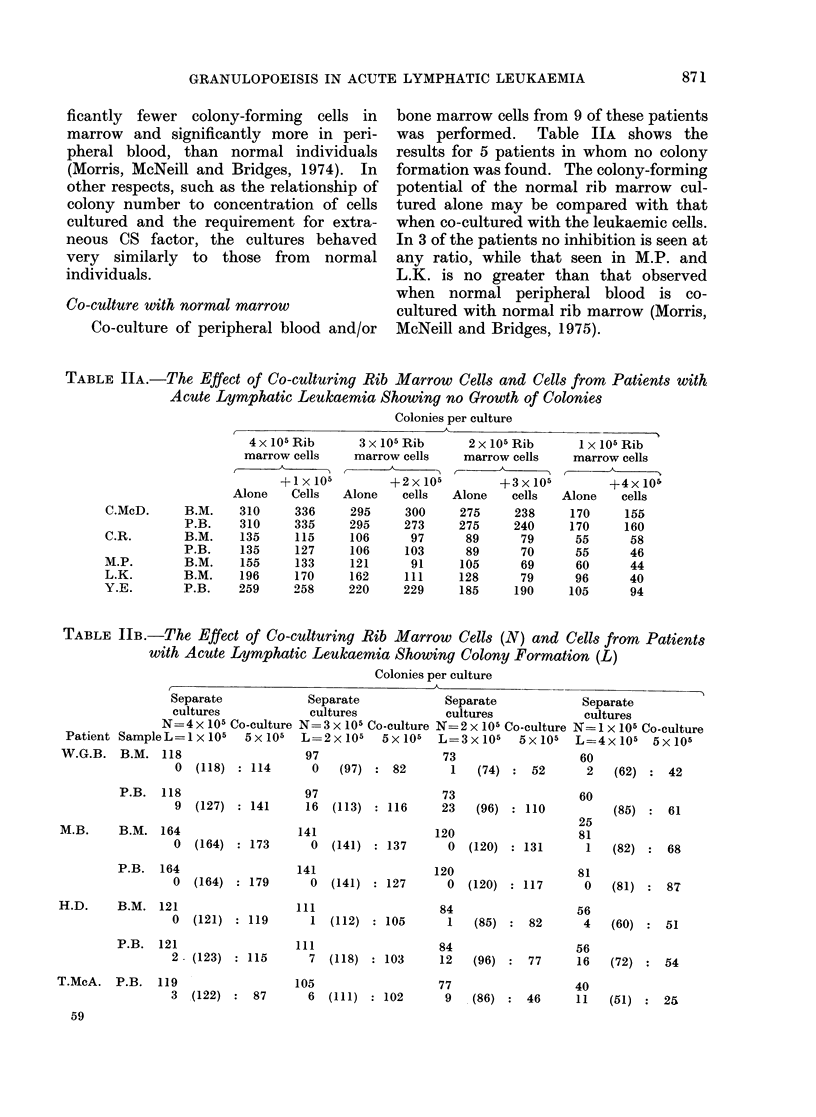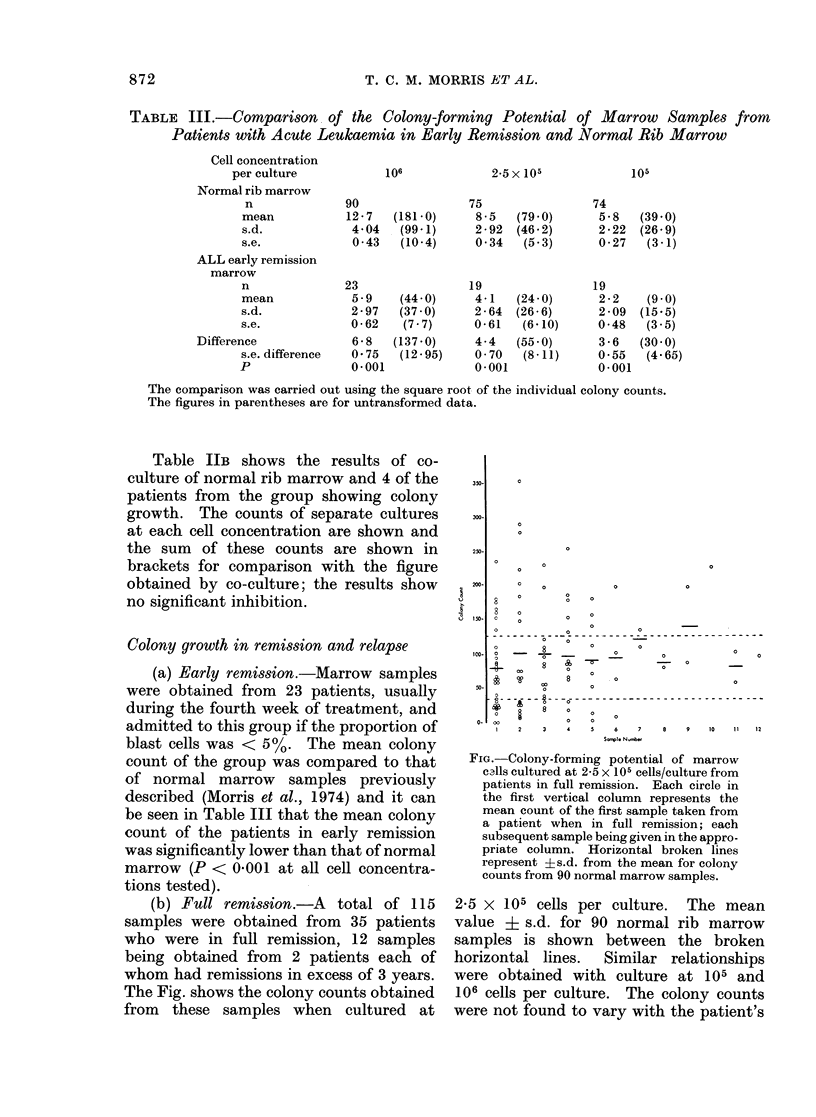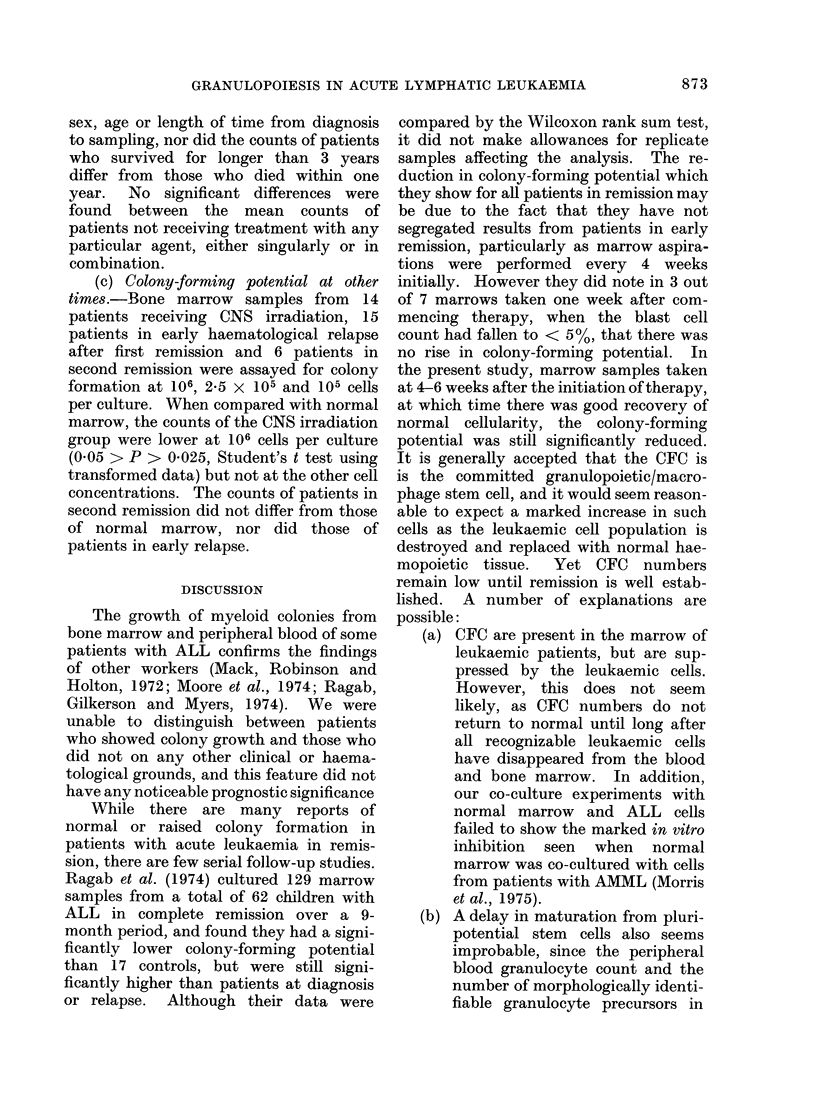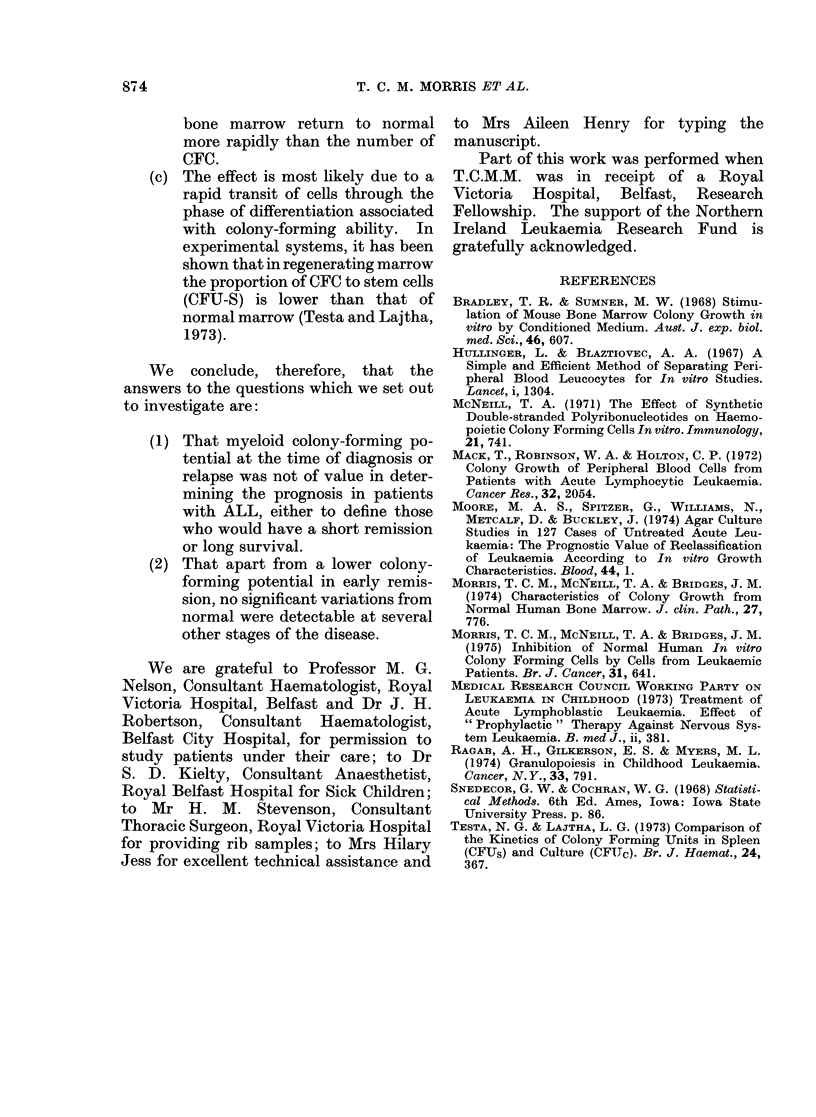# Changes in granulopoiesis detected by in vitro colony formation in acute lymphatic leukaemia.

**DOI:** 10.1038/bjc.1977.130

**Published:** 1977-06

**Authors:** T. C. Morris, M. Butler, J. G. Muldrew, T. A. McNeill, J. M. Bridges

## Abstract

Patients with acute lymphatic leukaemia (ALL) could be divided into two groups at diagnosis--those whose peripheral blood and/or bone marrow exhibited in vitro colony formation and those in whom it did not, but this finding did not appear to correlate with any clinical or haematological parameter, or with prognosis.The colonly-forming potential of patients with ALL in their first full remission, early relapse or second remission did not deviate significantly from previously established normal values, but the colony-forming potential of patients in early remission was very significantly reduced. No loss of colony-forming potential of normal marrow cells was noted when they were cultured with cells from patients with ALL.


*Br. J. Cancer* (1977) **35**, 868.

### CHANGES IN GRANULOPOIESIS DETECTED BY *IN VITRO* COLONY FORMATION IN ACUTE LYMPHATIC LEUKAEMIA

T. C. M. MORRIS[1], M. BUTLER[1], J. G. MULDREW[1], T. A. McNEILL[2] AND J. M. BRIDGES[3]

*From the [1]Department of Haematology, Royal Victoria Hospital; [2]Department of Microbiology, The Queen's University and [3]Royal Belfast Hospital for Sick Children, Belfast*

Received 8 November 1976 Accepted 25 January 1977

**Summary.—Patients with acute lymphatic leukaemia (ALL) could be divided into two groups at diagnosis—those whose peripheral blood and/or bone marrow exhibited *in vitro* colony formation and those in whom it did not, but this finding did not appear to correlate with any clinical or haematological parameter, or with prognosis. The colony-forming potential of patients with ALL in their first full remission, early relapse or second remission did not deviate significantly from previously established normal values, but the colony-forming potential of patients in early remission was very significantly reduced. No loss of colony-forming potential of normal marrow cells was noted when they were cultured with cells from patients with ALL.**

WHILE acute leukaemia in children is usually a disease of lymphatic origin, its nature is such that granulopoiesis is frequently compromised. Since the recovery and maintenance of granulocyte number and function is central to the achievement and maintenance of remission, the study of granulocyte precursors is relevant in ALL. One method for the assessment of granulocytic potential is the *in vitro* agar colony-forming cell (CFC) assay, in which colonies of granulocytes and macrophages may be grown from their common precursor cell (the CFC).

We have studied a total of 43 patients at various stages of their illness, bone marrow samples being taken only when these were indicated under the appropriate treatment protocol or as part of the general management of the patient. We have attempted to answer two basic questions:

(1) Certain prognostic factors are recognized in ALL, such as age, peripheral blood leucocyte count and mediastinal involvement, but they give only a crude guide to prognosis. Could the assessment of colony-forming potential or other features of colony growth help to distinguish patients with good or poor prognosis?

(2) What variations may be detected by the *in vitro* CFC assay during the various stages of the disease?

### MATERIALS AND METHODS

*Patients with leukaemia.*—Forty-three patients with acute lymphatic leukaemia were studied. Diagnosis was based on clinical and morphological grounds and, with the exception of 4 adults, all were accepted into the MRC CONCORD or UKALL trials (Medical Research Council Working Party, 1973) and treated according to the allocated protocol. The criteria for the diagnosis of remission and relapse were in accordance with the appropriate trial.

*Collection of samples.*—Samples of iliac crest marrow were aspirated under ketamine anaesthesia (local anaesthesia in older patients) and placed in bottles containing 5 ml collecting medium (BHK Eagle's from Wellcome Reagents Ltd) supplemented with 10% foetal calf serum (Flow Laboratories Ltd) and 10% trypticase soy broth (Difco), with 100

i.u. preservative-free heparin (Weddel Pharmaceuticals Ltd, London). Excess erythrocytes were removed by layering these samples over methyl-cellulose/triosil (Hullinger and Blaztiovec, 1967) and allowing them to sediment at room temperature for 30–50 min. The leucocyte-rich upper layer was collected, the leucocytes washed once, resuspended in collecting medium and a nucleated-cell count performed.

Peripheral blood samples from the patients were collected in preservative-free heparin and leucocyte suspensions prepared by allowing the blood to sediment and removing the leucocyte-rich supernatant plasma, the cells were concentrated by centrifugation, washed twice and resuspended in collecting medium and a nucleated-cell count performed prior to culture.

*Culture technique.*—All cultures were performed by the double-layer technique in Nunclon 30-mm plastic dishes (A/S Nunc, Denmark) using the modified Eagle's medium previously described (McNeill, 1971). CS factor was provided by the inclusion of 5% (v/v) of human spleen or human embryo cell-conditioned medium (Bradley and Sumner, 1968) in the Eagle's 1·2% agar underlayer, this being the optimum concentration for colony growth of normal human marrow, as determined by previous titration. Eagle's 0·3% agar medium was held at 37°C, cells added to the concentration required and 1-ml aliquots placed upon the gelled underlayers. All cell suspensions were cultured in quadruplicate. Cultures were incubated for 7 days at 37°C in sealed boxes containing 10% $CO_2$ in humidified air, and colonies (aggregates of $> 20$ cells) counted with a stereoscopic microscope at $\times 40$ magnification; the figures shown for colony counts are the mean of the 4 replicate cultures.

### *Co-culture procedure*

Normal bone marrow cells for co-culture were obtained from segments of rib removed at thoracotomy from patients in whom no haematological abnormality was present. The rib segments were placed in collecting medium and the cells suspended in this medium by washing through the medullary cavity with a Sahli marrow aspiration needle attached to a syringe.

The normal and leukaemic samples for co-culture were cultured separately at cell concentrations of 1, 2, 3 and 4 $\times 10^5$ cells per ml respectively. Co-cultures were performed so that the total number of cells per ml was kept constant at $5 \times 10^5$ providing cultures with ratios of 1 : 4, 2 : 3, 3 : 2 and 4 : 1 of the two cell populations being co-cultured. Where insufficient cells were available, all cell concentrations were halved, giving a total cell concentration of $2{\cdot}5 \times 10^5$ per ml.

### *Plan of study*

Samples were obtained from patients at 6 distinct phases of their illness. These were:

(i) At diagnosis.

(ii) During early remission, *i.e.*, leukaemic cells absent from peripheral blood and bone marrow, and before the start of prophylactic CNS irradiation (usually Week 4–6).

(iii) During full remission, *i.e.*, once cyclical maintenance therapy commenced. As many as 12 samples were obtained from some of the patients with prolonged remissions. In order to prevent statistical bias from the inclusion of a large number of results from one or two patients, the samples were arranged in subgroups for analysis, each subgroup containing only one sample from each patient. When a second sample had been cultured, this was entered into the second subgroup, and so on to the 12th subgroup. As samples from patients in remission were normally taken at 12-weekly intervals, the latter subgroups clearly contain the patients with prolonged remissions.

(iv) During the course of prophylactic CNS irradiation.

(v) Early relapse. These were patients in their first remission in whom bone marrow aspiration revealed an increase in blast cells.

(vi) Patients in second remission.

### *Statistical methods*

Tests of normality (Snedecor and Cochran, 1968) showed that the square roots of the colony counts were always normally distributed, although the untransformed data were not always so. The square roots of the colony counts were therefore used for comparison of the mean colony counts (and *t* tests) and for tests of correlation. These were performed on an Olivetti Program 101

Computer. Chi-squared tests for the comparison of two proportions were not applicable in some instances, because of small numbers in one portion of the table. Fisher's exact probability tests were therefore used and these were performed with an ICL 1907 computer.

### RESULTS

### *Colony growth at diagnosis*

Peripheral blood and/or bone marrow samples were obtained from 20 patients at diagnosis. Two distinct groups were found—8 patients whose cells grew colonies and 12 whose cells did not. These results, together with clinical and haematological data, are given in Tables Ia and Ib.

No difference between the two groups was found in terms of age, sex, peripheral blood leucocyte or blast-cell count. The degree of marrow replacement by leukaemic cells was similar, as was the degree of splenic and lymph-node involvement. Prognosis was also unaffected, since:

(a) all patients came into remission (except one who died from a cerebral haemorrhage) and
(b) median survival was similar in both groups (approximately 2 years) with both groups containing some long survivors.

It is of interest that those patients who possessed colony-forming ability had signi-

TABLE Ia.—*Clinical and Haematological Data of Patients whose Blood or Bone Marrow Grow Colonies at Diagnosis*

| Patient data | | | Peripheral blood leucocyte count (units) | % Blast cells | Degree of bone marrow replacement | Sample cultured | Colonies per culture of cell concentration | | | |
|---|---|---|---|---|---|---|---|---|---|---|
| Patient | Sex | Age | | | | | $1\times10^6$ | $5\times10^5$ | $2{\cdot}5\times10^5$ | $1\times10^5$ |
| W.G.B. | M | 4 | 6·0 | 83 | C* | P.B.† | 54±6 | 31±2 | 17±4 | 9±3 |
| | | | | | | B.M. | 7±1 | 2±1 | 1±1 | 0 |
| H.D. | F | 5 | 5·5 | 67 | C | P.B. | 44±1 | 20±2 | — | 2±1 |
| | | | | | | B.M. | 12±2 | 8±2 | — | 0 |
| M.McC. | M | 2 | 154·0 | 96 | C | P.B. | 3±2 | 0 | 0 | 0 |
| | | | | | | B.M. | 33±4 | — | 0 | 0 |
| T.McA. | M | 19 | 5·8 | 22 | C | P.B. | 19±3 | 15±2 | 8±2 | 3±1 |
| J.McB. | M | 44 | 6·3 | 49 | C | P.B. | 39±4 | — | — | 5±1 |
| F.D. | M | 45 | 11·4 | 68 | C | P.B. | 36±1 | 10±1 | 3±2 | 1±1 |
| P.M. | M | 5 | 1·7 | 7 | 30% | B.M. | 87±11 | 41±3 | — | — |
| M.B. | F | 6 | 7·0 | 57 | C | B.M. | 3±1 | 1±1 | — | 1±1 |

* C = Virtually complete replacement of marrow by leukaemic cells.
† P.B. = peripheral blood: B.M.; bone marrow.

TABLE Ib.—*Clinical and Haematological Data of Patients whose Blood and Bone Marrow did not Grow Colonies at Diagnosis*

| Patient data | | | Peripheral blood leucocyte count (units) | % Blast cells | Degree of bone marrow replacement |
|---|---|---|---|---|---|
| Patient | Sex | Age | | | |
| C.R. | M | 12 | 100·7 | 94 | C* |
| D.B. | M | 12 | 26·3 | 83 | C |
| C.McD. | M | 5 | 73·5 | 80 | C |
| M.McA. | F | 8 | 13·6 | 68 | 80% |
| | | | 17·7 | 80 | C |
| Y.E. | F | 33 | 512·0 | 90 | — |
| M.P. | M | 3 | 4·7 | 52 | C |
| L.K. | F | 3 | 27·6 | 80 | C |
| S.P. | F | 6 | 1·8 | 70 | C |
| J.McC. | M | 17 | 0·4 | 4 | C |
| L.McG. | M | 8 | 3·9 | 33 | 70% |
| D.McD. | F | 4 | 55·2 | 85 | C |
| G.M. | M | 5 | 2·7 | 48 | C |

* C = Virtually complete replacement of marrow by leukaemic cells.

ficantly fewer colony-forming cells in marrow and significantly more in peripheral blood, than normal individuals (Morris, McNeill and Bridges, 1974). In other respects, such as the relationship of colony number to concentration of cells cultured and the requirement for extraneous CS factor, the cultures behaved very similarly to those from normal individuals.

*Co-culture with normal marrow*

Co-culture of peripheral blood and/or bone marrow cells from 9 of these patients was performed. Table IIA shows the results for 5 patients in whom no colony formation was found. The colony-forming potential of the normal rib marrow cultured alone may be compared with that when co-cultured with the leukaemic cells. In 3 of the patients no inhibition is seen at any ratio, while that seen in M.P. and L.K. is no greater than that observed when normal peripheral blood is co-cultured with normal rib marrow (Morris, McNeill and Bridges, 1975).

TABLE IIA.—*The Effect of Co-culturing Rib Marrow Cells and Cells from Patients with Acute Lymphatic Leukaemia Showing no Growth of Colonies*

| | | Colonies per culture | | | | | | | |
|---|---|---|---|---|---|---|---|---|---|
| | | $4\times10^5$ Rib marrow cells | | $3\times10^5$ Rib marrow cells | | $2\times10^5$ Rib marrow cells | | $1\times10^5$ Rib marrow cells | |
| | | Alone | $+1\times10^5$ Cells | Alone | $+2\times10^5$ cells | Alone | $+3\times10^5$ cells | Alone | $+4\times10^5$ cells |
| C.McD. | B.M. | 310 | 336 | 295 | 300 | 275 | 238 | 170 | 155 |
| | P.B. | 310 | 335 | 295 | 273 | 275 | 240 | 170 | 160 |
| C.R. | B.M. | 135 | 115 | 106 | 97 | 89 | 79 | 55 | 58 |
| | P.B. | 135 | 127 | 106 | 103 | 89 | 70 | 55 | 46 |
| M.P. | B.M. | 155 | 133 | 121 | 91 | 105 | 69 | 60 | 44 |
| L.K. | B.M. | 196 | 170 | 162 | 111 | 128 | 79 | 96 | 40 |
| Y.E. | P.B. | 259 | 258 | 220 | 229 | 185 | 190 | 105 | 94 |

TABLE IIB.—*The Effect of Co-culturing Rib Marrow Cells (N) and Cells from Patients with Acute Lymphatic Leukaemia Showing Colony Formation (L)*

| | | Colonies per culture | | | | | | | |
|---|---|---|---|---|---|---|---|---|---|
| Patient | Sample | Separate cultures $N=4\times10^5$ $L=1\times10^5$ | Co-culture $5\times10^5$ | Separate cultures $N=3\times10^5$ $L=2\times10^5$ | Co-culture $5\times10^5$ | Separate cultures $N=2\times10^5$ $L=3\times10^5$ | Co-culture $5\times10^5$ | Separate cultures $N=1\times10^5$ $L=4\times10^5$ | Co-culture $5\times10^5$ |
| W.G.B. | B.M. | 118 | | 97 | | 73 | | 60 | |
| | | 0 | (118) : 114 | 0 | (97) : 82 | 1 | (74) : 52 | 2 | (62) : 42 |
| | P.B. | 118 | | 97 | | 73 | | 60 | |
| | | 9 | (127) : 141 | 16 | (113) : 116 | 23 | (96) : 110 | 25 | (85) : 61 |
| M.B. | B.M. | 164 | | 141 | | 120 | | 81 | |
| | | 0 | (164) : 173 | 0 | (141) : 137 | 0 | (120) : 131 | 1 | (82) : 68 |
| | P.B. | 164 | | 141 | | 120 | | 81 | |
| | | 0 | (164) : 179 | 0 | (141) : 127 | 0 | (120) : 117 | 0 | (81) : 87 |
| H.D. | B.M. | 121 | | 111 | | 84 | | 56 | |
| | | 0 | (121) : 119 | 1 | (112) : 105 | 1 | (85) : 82 | 4 | (60) : 51 |
| | P.B. | 121 | | 111 | | 84 | | 56 | |
| | | 2 | (123) : 115 | 7 | (118) : 103 | 12 | (96) : 77 | 16 | (72) : 54 |
| T.McA. | P.B. | 119 | | 105 | | 77 | | 40 | |
| | | 3 | (122) : 87 | 6 | (111) : 102 | 9 | (86) : 46 | 11 | (51) : 25 |

TABLE III.—*Comparison of the Colony-forming Potential of Marrow Samples from Patients with Acute Leukaemia in Early Remission and Normal Rib Marrow*

| Cell concentration per culture | $10^6$ | | $2 \cdot 5 \times 10^5$ | | $10^5$ | |
|---|---|---|---|---|---|---|
| Normal rib marrow | | | | | | |
| n | 90 | | 75 | | 74 | |
| mean | 12·7 | (181·0) | 8·5 | (79·0) | 5·8 | (39·0) |
| s.d. | 4·04 | (99·1) | 2·92 | (46·2) | 2·22 | (26·9) |
| s.e. | 0·43 | (10·4) | 0·34 | (5·3) | 0·27 | (3·1) |
| ALL early remission marrow | | | | | | |
| n | 23 | | 19 | | 19 | |
| mean | 5·9 | (44·0) | 4·1 | (24·0) | 2·2 | (9·0) |
| s.d. | 2·97 | (37·0) | 2·64 | (26·6) | 2·09 | (15·5) |
| s.e. | 0·62 | (7·7) | 0·61 | (6·10) | 0·48 | (3·5) |
| Difference | 6·8 | (137·0) | 4·4 | (55·0) | 3·6 | (30·0) |
| s.e. difference | 0·75 | (12·95) | 0·70 | (8·11) | 0·55 | (4·65) |
| *P* | 0·001 | | 0·001 | | 0·001 | |

The comparison was carried out using the square root of the individual colony counts. The figures in parentheses are for untransformed data.

Table IIB shows the results of co-culture of normal rib marrow and 4 of the patients from the group showing colony growth. The counts of separate cultures at each cell concentration are shown and the sum of these counts are shown in brackets for comparison with the figure obtained by co-culture; the results show no significant inhibition.

*Colony growth in remission and relapse*

(a) *Early remission.*—Marrow samples were obtained from 23 patients, usually during the fourth week of treatment, and admitted to this group if the proportion of blast cells was < 5%. The mean colony count of the group was compared to that of normal marrow samples previously described (Morris *et al.*, 1974) and it can be seen in Table III that the mean colony count of the patients in early remission was significantly lower than that of normal marrow ($P < 0 \cdot 001$ at all cell concentrations tested).

(b) *Full remission.*—A total of 115 samples were obtained from 35 patients who were in full remission, 12 samples being obtained from 2 patients each of whom had remissions in excess of 3 years. The Fig. shows the colony counts obtained from these samples when cultured at

FIG.—Colony-forming potential of marrow cells cultured at $2 \cdot 5 \times 10^5$ cells/culture from patients in full remission. Each circle in the first vertical column represents the mean count of the first sample taken from a patient when in full remission; each subsequent sample being given in the appropriate column. Horizontal broken lines represent ±s.d. from the mean for colony counts from 90 normal marrow samples.

$2 \cdot 5 \times 10^5$ cells per culture. The mean value ± s.d. for 90 normal rib marrow samples is shown between the broken horizontal lines. Similar relationships were obtained with culture at $10^5$ and $10^6$ cells per culture. The colony counts were not found to vary with the patient's sex, age or length of time from diagnosis to sampling, nor did the counts of patients who survived for longer than 3 years differ from those who died within one year. No significant differences were found between the mean counts of patients not receiving treatment with any particular agent, either singularly or in combination.

(c) *Colony-forming potential at other times.*—Bone marrow samples from 14 patients receiving CNS irradiation, 15 patients in early haematological relapse after first remission and 6 patients in second remission were assayed for colony formation at $10^6$, $2{\cdot}5 \times 10^5$ and $10^5$ cells per culture. When compared with normal marrow, the counts of the CNS irradiation group were lower at $10^6$ cells per culture ($0{\cdot}05 > P > 0{\cdot}025$, Student's $t$ test using transformed data) but not at the other cell concentrations. The counts of patients in second remission did not differ from those of normal marrow, nor did those of patients in early relapse.

### DISCUSSION

The growth of myeloid colonies from bone marrow and peripheral blood of some patients with ALL confirms the findings of other workers (Mack, Robinson and Holton, 1972; Moore *et al.*, 1974; Ragab, Gilkerson and Myers, 1974). We were unable to distinguish between patients who showed colony growth and those who did not on any other clinical or haematological grounds, and this feature did not have any noticeable prognostic significance

While there are many reports of normal or raised colony formation in patients with acute leukaemia in remission, there are few serial follow-up studies. Ragab *et al.* (1974) cultured 129 marrow samples from a total of 62 children with ALL in complete remission over a 9-month period, and found they had a significantly lower colony-forming potential than 17 controls, but were still significantly higher than patients at diagnosis or relapse. Although their data were compared by the Wilcoxon rank sum test, it did not make allowances for replicate samples affecting the analysis. The reduction in colony-forming potential which they show for all patients in remission may be due to the fact that they have not segregated results from patients in early remission, particularly as marrow aspirations were performed every 4 weeks initially. However they did note in 3 out of 7 marrows taken one week after commencing therapy, when the blast cell count had fallen to $< 5\%$, that there was no rise in colony-forming potential. In the present study, marrow samples taken at 4–6 weeks after the initiation of therapy, at which time there was good recovery of normal cellularity, the colony-forming potential was still significantly reduced. It is generally accepted that the CFC is is the committed granulopoietic/macrophage stem cell, and it would seem reasonable to expect a marked increase in such cells as the leukaemic cell population is destroyed and replaced with normal haemopoietic tissue. Yet CFC numbers remain low until remission is well established. A number of explanations are possible:

(a) CFC are present in the marrow of leukaemic patients, but are suppressed by the leukaemic cells. However, this does not seem likely, as CFC numbers do not return to normal until long after all recognizable leukaemic cells have disappeared from the blood and bone marrow. In addition, our co-culture experiments with normal marrow and ALL cells failed to show the marked *in vitro* inhibition seen when normal marrow was co-cultured with cells from patients with AMML (Morris *et al.*, 1975).

(b) A delay in maturation from pluripotential stem cells also seems improbable, since the peripheral blood granulocyte count and the number of morphologically identifiable granulocyte precursors in bone marrow return to normal more rapidly than the number of CFC.

(c) The effect is most likely due to a rapid transit of cells through the phase of differentiation associated with colony-forming ability. In experimental systems, it has been shown that in regenerating marrow the proportion of CFC to stem cells (CFU-S) is lower than that of normal marrow (Testa and Lajtha, 1973).

We conclude, therefore, that the answers to the questions which we set out to investigate are:

(1) That myeloid colony-forming potential at the time of diagnosis or relapse was not of value in determining the prognosis in patients with ALL, either to define those who would have a short remission or long survival.

(2) That apart from a lower colony-forming potential in early remission, no significant variations from normal were detectable at several other stages of the disease.

We are grateful to Professor M. G. Nelson, Consultant Haematologist, Royal Victoria Hospital, Belfast and Dr J. H. Robertson, Consultant Haematologist, Belfast City Hospital, for permission to study patients under their care; to Dr S. D. Kielty, Consultant Anaesthetist, Royal Belfast Hospital for Sick Children; to Mr H. M. Stevenson, Consultant Thoracic Surgeon, Royal Victoria Hospital for providing rib samples; to Mrs Hilary Jess for excellent technical assistance and to Mrs Aileen Henry for typing the manuscript.

Part of this work was performed when T.C.M.M. was in receipt of a Royal Victoria Hospital, Belfast, Research Fellowship. The support of the Northern Ireland Leukaemia Research Fund is gratefully acknowledged.

### REFERENCES

BRADLEY, T. R. & SUMNER, M. W. (1968) Stimulation of Mouse Bone Marrow Colony Growth *in vitro* by Conditioned Medium. *Aust. J. exp. biol. med. Sci.*, **46**, 607.

HULLINGER, L. & BLAZTIOVEC, A. A. (1967) A Simple and Efficient Method of Separating Peripheral Blood Leucocytes for *In vitro* Studies. *Lancet*, i, 1304.

MCNEILL, T. A. (1971) The Effect of Synthetic Double-stranded Polyribonucleotides on Haemopoietic Colony Forming Cells *In vitro*. *Immunology*, **21**, 741.

MACK, T., ROBINSON, W. A. & HOLTON, C. P. (1972) Colony Growth of Peripheral Blood Cells from Patients with Acute Lymphocytic Leukaemia. *Cancer Res.*, **32**, 2054.

MOORE, M. A. S., SPITZER, G., WILLIAMS, N., METCALF, D. & BUCKLEY, J. (1974) Agar Culture Studies in 127 Cases of Untreated Acute Leukaemia: The Prognostic Value of Reclassification of Leukaemia According to *In vitro* Growth Characteristics. *Blood*, **44**, 1.

MORRIS, T. C. M., MCNEILL, T. A. & BRIDGES, J. M. (1974) Characteristics of Colony Growth from Normal Human Bone Marrow. *J. clin. Path.*, **27**, 776.

MORRIS, T. C. M., MCNEILL, T. A. & BRIDGES, J. M. (1975) Inhibition of Normal Human *In vitro* Colony Forming Cells by Cells from Leukaemic Patients. *Br. J. Cancer*, **31**, 641.

MEDICAL RESEARCH COUNCIL WORKING PARTY ON LEUKAEMIA IN CHILDHOOD (1973) Treatment of Acute Lymphoblastic Leukaemia. Effect of "Prophylactic" Therapy Against Nervous System Leukaemia. *B. med J.*, ii, 381.

RAGAB, A. H., GILKERSON, E. S. & MYERS, M. L. (1974) Granulopoiesis in Childhood Leukaemia. *Cancer, N.Y.*, **33**, 791.

SNEDECOR, G. W. & COCHRAN, W. G. (1968) *Statistical Methods*. 6th Ed. Ames, Iowa: Iowa State University Press. p. 86.

TESTA, N. G. & LAJTHA, L. G. (1973) Comparison of the Kinetics of Colony Forming Units in Spleen ($CFU_S$) and Culture ($CFU_C$). *Br. J. Haemat.*, **24**, 367.